# A Core Invasiveness Gene Signature Reflects Epithelial-to-Mesenchymal Transition but Not Metastatic Potential in Breast Cancer Cell Lines and Tissue Samples

**DOI:** 10.1371/journal.pone.0089262

**Published:** 2014-02-21

**Authors:** Melike Marsan, Gert Van den Eynden, Ridha Limame, Patrick Neven, Jan Hauspy, Peter A. Van Dam, Ignace Vergote, Luc Y. Dirix, Peter B. Vermeulen, Steven J. Van Laere

**Affiliations:** 1 Translational Cancer Research Unit, Oncology Center, GZA Hospitals Sint-Augustinus, Antwerp, Belgium; 2 Laboratory for Cancer Research and Clinical Oncology, University of Antwerp, Antwerp, Belgium; 3 Department of oncology, KU Leuven, Leuven, Belgium; 4 Gynaecologic oncology, UZA, Antwerp, Belgium; Institute of Molecular and Cell Biology, Biopolis, United States of America

## Abstract

**Introduction:**

Metastases remain the primary cause of cancer-related death. The acquisition of invasive tumour cell behaviour is thought to be a cornerstone of the metastatic cascade. Therefore, gene signatures related to invasiveness could aid in stratifying patients according to their prognostic profile. In the present study we aimed at identifying an invasiveness gene signature and investigated its biological relevance in breast cancer.

**Methods & Results:**

We collected a set of published gene signatures related to cell motility and invasion. Using this collection, we identified 16 genes that were represented at a higher frequency than observed by coincidence, hereafter named the core invasiveness gene signature. Principal component analysis showed that these overrepresented genes were able to segregate invasive and non-invasive breast cancer cell lines, outperforming sets of 16 randomly selected genes (all P<0.001). When applied onto additional data sets, the expression of the core invasiveness gene signature was significantly elevated in cell lines forced to undergo epithelial-mesenchymal transition. The link between core invasiveness gene expression and epithelial-mesenchymal transition was also confirmed in a dataset consisting of 2420 human breast cancer samples. Univariate and multivariate Cox regression analysis demonstrated that CIG expression is not associated with a shorter distant metastasis free survival interval (HR = 0.956, 95%C.I. = 0.896–1.019, P = 0.186).

**Discussion:**

These data demonstrate that we have identified a set of core invasiveness genes, the expression of which is associated with epithelial-mesenchymal transition in breast cancer cell lines and in human tissue samples. Despite the connection between epithelial-mesenchymal transition and invasive tumour cell behaviour, we were unable to demonstrate a link between the core invasiveness gene signature and enhanced metastatic potential.

## Introduction

Breast cancer is the leading cause of cancer-related death amongst women worldwide [1]. In most cases, it is not the primary tumour that is lethal but the development of distant metastases. In order to metastasize, tumour cells need to break away from the primary site to bridge the gap with the surrounding lymph or blood vessels. Once blood borne, the tumour cells usurp the bloodstream to passively reach distant organs where they extravasate to form metastatic deposits. Numerous biological processes including cell motility, the acquisition of an invasive phenotype by cancer cells, angiogenesis and anti-apoptosis orchestrate the metastatic process [2] [3].

One of the first steps of the metastatic cascade is the acquisition of a motile and invasive phenotype by cancer cells. Recently, it has been recognized that cancer cell invasion is a heterogeneous process covering at least five distinct patterns including rounded/amoeboid migration, Epithelial-Mesenchymal Transition (EMT) driven migration, multicellular streaming, collective invasion and expansive growth [4]. Only the latter pattern is a passive process in which cancer cells invade the surrounding tissue as a consequence of being pushed by the expanding body of the tumour. All other patterns require a certain degree of plasticity allowing cancer cells to adapt to diverse structural, molecular and even adverse microenvironmental conditions. In addition, cancer cells are allowed to switch between different invasive patterns as the microenvironmental conditions change along their journey, leading to the existence of transition states that further extravagate the complexity of the process [4].

The dynamic behaviour of cancer cells during invasion is, underpinned by changes in the expression of multiple genes, both inside the cancer cells and in host cells residing in the surrounding stroma. These genes can be regarded as biomarkers to monitor the presence of these invasive cell populations in human samples. The identification of such biomarkers has potential clinical value, as they might assist in the identification of patients with a higher propensity of developing distant metastases. Also, the search for biomarkers can result in the identification of novel targets for therapy. In case of cancer cell invasion, blocking such targets might lead to the confinement of the primary tumour to its original site, reducing cancer to a local and more curable problem. However, due to complex biology of cancer cell invasion, identifying such biomarkers is a daunting task.

The present study aims at identifying biomarkers for cancer cell invasion by taking advantage of a collection of recently published gene signatures specific for invasive or motile cells derived through genome-wide gene expression profiling [5]–[22]. Given the high frequency of false positive results associated with this kind of experiments, we hypothesize that genes represented multiple times in these profiles have a higher propensity of being true biomarkers for tumour cell motility and invasion as compared to genes identified only once. The identified biomarker panel was validated using a series of *in silico* experiments and its translational relevance was analysed using a collection of publicly available gene expression profiles derived from approximately 2500 breast tumour samples.

## Materials and Methods

### Gene Selection

In order to identify a set of marker genes related to invasion, we adopted the following strategy. We reviewed the literature in search for studies reporting on gene expression profiles of motile or invasive cells, not necessarily related to cancer. The included gene signatures and their corresponding references are summarized in **Table 1**. Two gene signatures were generated using publicly available data sets (GSE11279 and GSE12917) **(Figure S1)**. To allow for cross-study comparability we translated the gene identifiers into gene symbols. Next we performed an overrepresentation analysis to identify genes that were reported 2, 3, 4, 5 and so on times across different studies. We reason that genes represented multiple times have a higher propensity of being true biomarkers of tumour cell invasion. To evaluate the significance of the overrepresentation we first identified the invasiveness gene universe, which is composed of all genes that have been reported in the collection of invasion-related gene signatures. Next, we generated gene lists by randomly selecting genes from the gene universe. The number of the random gene lists equalled the number of the gene lists in the original collection. Also, the number of genes included in the random gene lists matched the number of genes included in the original gene signatures. Finally, we identified the genes that were selected 2, 3, 4, 5 and so on times in the collection of random gene lists. As such, we obtained expected overrepresentation frequencies and compared those to the observed overrepresentation frequencies using a Chi-square test. The list of biomarkers related to tumour cell invasion, hereafter termed Core Invasiveness Gene (CIG) signature, is composed of those genes for which the observed overrepresentation frequency surpassed the expected overrepresentation frequency given a significant P-value for the Chi-square test.

**Table 1:** Collection of gene signatures used for overrepresentation analysis.

	Description Gene Signature	# Genes	% Original	Reference
1.	Differentially expressed genes by TGFβ in p53-depleted MDA-MB-231 cells	105	5	Adorno et al. 2009 [5]
2.	Coculture of mesenchymal stromal cells with CD133+ hematopoietic stem cells	21	2	Alakel et al. 2009 [6]
3.	TGFβ-induced EMT in HMECs predisposed to ionizing radiation	32	3	Andarawewa et al. 2007 [7]
4.	Trophoblast invasion-related genes	648	7	Bilban et al. 2009 [5]
5.	Overexpression of Integrin α6β4 in MDA-MB-435 cells	263	10	Chen et al. 2009 [5]
6.	Ezrin knockdown in SW480	26	2	GSE11297
7.	Comparison of MDA-MB-231 cells with wild-type SNAIL and dominant negative SNAIL	50	2	Fabre-Guillevin et al. 2008 [10]
8.	Comparison of MDA-MB-435 cells with wild-type NM23-H1 and mutant NM23-H1	44	3	Horak et al. 2007 [11]
9.	Functional implications of non-lens βγ-Cristallin and Refoil Factor Complex	55	6	Liu et al. 2008 [12]
10.	Gene expression profiling of central and peripheral zones of pancreatic carcinoma	756	12	Nakamura et al. 2007 [13]
11.	Normal HMECs vs. HMECs transfected with constitutively active RhoA	135	2	GSE12917
12.	Overexpression of classIIb HLH factors E2-2A and E2-2B in MDCK cells	147	5	Sobrado et al. 2009 [14]
13.	Genes epigenetically regulated in poorly metastatic MDA-MB-468 cells vs the highly metastatic MDA-MB-468LN variant	136	2	Rodenhiser et al. 2008 [15]
14.	Expression profiling of migratory cells in the Drosophila ovary	33	3	Wang et al. 2006 [16]]
15.	Transfection of MIR-520C in MCF-7 cells	113	4	Huang et al. 2008 [17
16.	Transfection of MIR-373 in MCF-7 cells	128	7	Huang et al. 2008 [17
17.	Comparison of mesenchymal and epithelial cells	186	3	Choi et al. 2010 [18]
18.	Genes differentially expressed in mesenchymal stem cells induced by CCL25	105	3	Binger et al. 2009 [19]
19.	Genes differentially expressed across a collection of 10 migratory glioma cell lines	89	1	Demuth et al. 2009 [20]
20.	Genes differentially expressed in MDA-MB-231 cells after CD146 downmodulation	45	3	Zabouo et al. 2009 [21]
21.	Keratinocyts treated with TGFβ to suppress proliferation but not migration	92	4	Cheng et al. 2008 [22]

### Validation of the CIG Signature

To validate the CIG signature we downloaded 3 data sets of gene expression profiles of breast cancer cell lines (Gene Expression Omnibus: GSE12777 [23] and GSE16795 [24]; Array Express: E-TABM-157 [25]. Each expression data set was normalized using GCRMA and probe sets with a fluorescence intensity above log2(100) in at least 10% of the arrays were filtered in. In addition, we filtered out all probe sets with inconsistent expression data. Therefore, we adopted the following strategy using the breast cancer cell line data sets GSE12777, GSE16795 and E-TABM-157. First, we identified the cell lines commonly profiled in all three data sets (N = 21). Next, we calculated Spearman correlation coefficients for all common probe sets (22.277) between each pair of data sets, resulting in three correlation coefficients per probe set. Those probe sets with a median correlation coefficient less than 0.50 (N = 11.689) were considered inconsistent and were filtered out for further analysis.

The breast cancer cell lines were classified as invasive and non-invasive according to the data published by Neve et al [25]. An arbitrary cut-off value of 500 cells per 75.000 seeded cells in a modified Boyden chamber assay was chosen to determine the classification. The CIG signature was applied onto the data sets using principal component analysis (PCA). The centroid of the invasive and the non-invasive breast cancer cell lines on the 2D-scatter plot representation of the PCA was determined. The Euclidean distance between both centroids was calculated and its significance was assessed using class label permutation (N = 100).

To evaluate our hypothesis that genes identified multiple times across different studies are robust biomarkers of tumour cell invasion, we selected 100 random gene lists of equal length to the CIG signature from the invasiveness gene universe (*vide supra*). Each of these signatures was applied onto the data sets using PCA. The Euclidean distances between the centroids of invasive and the non-invasive breast cancer cell lines for the random gene lists were calculated and statistically compared with the Euclidean distance obtained using the CIG signature.

Finally, we evaluated the robustness of the individual CIGs by means of their regression coefficients for the first metagene in each data set. The sign and absolute value of the regression coefficients for each CIG was compared between the data sets. Similar values for both criteria are considered as evidence for robustness. The median values of these regression coefficients were used to calculate the CIG expression for new samples (*vide infra*).

### Association between CIG Expression, Epithelial-to-Mesenchymal Transition (EMT) and Metastatic Potential

To investigate the relationship between CIG expression and EMT we downloaded 2 data sets of time series of tumour cells forced to undergo EMT using either TGFβ alone (GSE17708) [26] or in combination with TNFα (GSE12548) [27]. To delineate EMT-driving mechanisms represented by the CIG signature we analysed a data set on HMLE cells (GSE24202), retrovirally transduced with vectors encoding EMT-inducing factors (TWIST, SNAI1, GSC, CDH1, TGFβ) [28]. To investigate the relationship between CIG expression and metastatic propensity we analysed a data set generated on 4T1-derived cell lines (GSE11259) [29]. The 4T1 tumour is a clinically relevant murine model of spontaneous breast cancer metastasis. The distinct 4T1-derived cell lines (4T1, 67NR and 66cl4) have been characterized for expression of EMT-features, *in vitro* invasiveness and *in vivo* metastatic ability in previous studies [29]. For all data sets, raw gene expression data were preprocessed as described before. CIG expression was calculated as described above using the informative genes only and compared between different groups using the Kruskal-Wallis test for multiple groups followed by post hoc testing (Tukey HSD) when appropriate. Changes in CIG expression in function of exposure time was evaluated using Spearman correlation coefficients.

### CIG Expression in Human Breast Cancer

To evaluate the biological significance of CIG expression in breast cancer, 12 data sets vouching for a total of 2420 human breast tumours were downloaded. The incorporated data sets [30]-[40] are summarized in **Table 2**. Each of these data sets was generated on the Affymetrix HGU133A platform. Raw expression data were normalized using the frozen RMA-algorithm to allow for cross-data set comparisons. Data preprocessing was done on the combined data set as described above resulting in 9.889 informative probe sets. Distant-metastases-free survival (DMFS) data were retrieved when available. CIG expression was calculated as described above.

**Table 2:** Gene expression data sets used throughout this study.

Group	ID	Repository	Platform	N	DMFS	Remark	Reference
***Breast cancer*** ***cell lines***	E-TABM-157	Array express	HGU133A	51	NA	Breast cancer cell line collection	Neve et al. 2006 [25]
	GSE12777	GEO	HGU133PLUS2	39	NA	Breast cancer cell line collection	Hollestelle et al. 2009 [24]
	GSE16795	GEO	HGU133A	51	NA	Breast cancer cell line collection	Hoeflich et al. 2009 [23]
	GSE11279	GEO	HGU133PLUS2	4	NA	Ezrin knockdown SW480	–
	GSE12917	GEO	HGU133PLUS2	6	NA	Normal and RhoA-transfectedHMECs	–
	GSE12548	GEO	HGU133PLUS2	20	NA	EMT time series in ARPE19	Takahashi et al. 2010 [27]
	GSE17708	GEO	HGU133PLUS2	26	NA	Time course of A549 treatedwith TGFβ	Sartor et al. 2010 [26]
	GSE24202	GEO	HGU133A	21	NA	HMLEs transfectedwith EMT-inducers	Taube et al. 2010 [28]
	GSE11259	GEO	HGU133v2	9	NA	[Non]Metastatic 4T1 clones	Lou et al. 2008 [29]
***Breast cancer*** ***patient samples***	GSE1456	GEO	HGU133A	159	–	None	Pawitan et al. 2005 [30]
	GSE2034	GEO	HGU133A	286	286	Lymph node negative cohort	Wang et al. 2005 [31]
	GSE2603	GEO	HGU133A	99	82	None	Minn et al. 2005 [32]
	GSE2990	GEO	HGU133A	189	125	Cohort used for generation of GGI	Sotiriou et al. 2006 [33]
	GSE4922	GEO	HGU133A	289	–	None	Ivshina et al. 2006 [34]
	GSE5327	GEO	HGU133A	58	58	ER negative cohort	Minn et al. 2007 [35]
	GSE7390	GEO	HGU133A	198	198	Lymph node negative cohort	Desmedt et al. 2007 [36]
	GSE11121	GEO	HGU133A	200	200	Lymph node negative cohort	Schmidt et al. 2008 [37]
	GSE12093	GEO	HGU133A	136	–	ER+, tamoxifen treated	Zhang et al. 2009 [38]
	GSE17705	GEO	HGU133A	298	298	ER+, tamoxifen treated	Symmans et al. 2010 [39]
	GSE25055	GEO	HGU133A	310	66	ErbB2-, Anthracyclin/Taxane-treated	Hatzis et al. 2011 [40]
	GSE25065	GEO	HGU133A	198	198	ErbB2-, Anthracyclin/Taxane-treated	Hatzis et al. 2011 [40]

Using the Single Sample Predictor (SSP)-algorithm [41] we classified the samples according to the molecular subtypes. The subtype-specific classification scores, cell proliferation scores and Risk-of-Relapse (ROR)-scores were retained for further analysis. Using correlation-based classifiers, each sample was classified according to the nine-cell line Claudin-low predictor [42], the wound healing response (WHR) signature [43], a stromal gene expression signature (STR) [44], the invasiveness gene signature (IGS) [45], the 70-gene prognostic signature (70G) [46], a classifier for CD44+ cells [47], a mammosphere-derived classifier [48] and the differentiation predictor model [49]. The pathway signatures described by Gatza and colleagues [50] and a VEGF-activation signature [51] were applied as outlined in the original manuscripts. To explore the link between deregulated activity of transcription factors involved in EMT on the one hand and CIG expression on the other hand we constructed and applied SNAIL, TWIST, GSC and E-Cadherin activation signatures. A TGFβ-specific gene signature was not constructed, as it was available from the publication by Gatza and colleagues [50]. Core-EMT metagene expression was calculated using the core-EMT signature genes reported by Taube and colleagues [28] **(Figure S2–Figure S3)**.

Signature classification scores, pathway activation scores and metagene expression data were subjected to unsupervised hierarchical cluster analysis with the Spearman correlation coefficient as distance measure and complete linkage as the dendrogram drawing method. Cox regression analysis was used to test for associations with DMFS data. ROR scores were not included as they were designed to predict relapse-free survival instead of DMFS. Multivariate analysis was performed in the backward setting and only variables significantly associated with DMFS in the univariate setting were included. Two multivariate models were constructed. In a first model, we analysed the gene signatures associated with the molecular subtypes, patient prognosis, tumour-associated stroma, pathway activation, stem cell biology and EMT separately. In a second model, the significant variables from the first model were included.

## Results

### Identification and Validation of the CIG Signature

**Figure 1:**
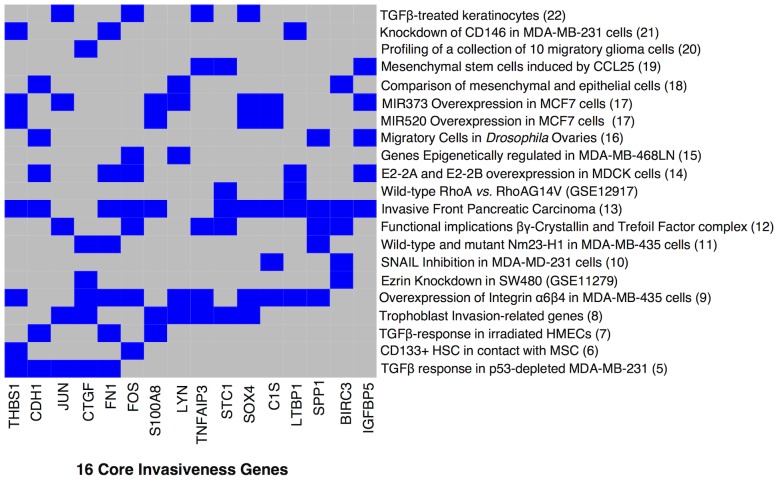
Core invasiveness genes/gene signature collection Interaction matrix. Interaction matrix representing the core invasiveness genes in the X-axis and the gene signature collection used for the overrepresentation analysis in the Y-axis. A blue cell indicates membership of the associated CIG in the corresponding gene signature. Most of the signatures count at least 2 CIGs in their gene lists except for the gene signature indentified in migratory glioma cells.

All gene signatures related to cell motility and invasion used in the overrepresentation analysis are provided in **Table 1**. All signatures combined represent a total of 2636 unique genes, from which 646, 202, 62, 16, 6 and 3 genes were represented 2, 3, 4, 5, 6 and 7 times respectively. We determined that the expected number of genes represented 2, 3, 4, 5, 6 and 7 times is respectively 1065, 376, 87, 14, 1 and 1. Both distributions were significantly different (Chi-Square test, P<0.001). The largest gene set for which the observed number exceeds the expected number was termed the Core Invasiveness Gene signature. The genes included in this signature are BIRC3, C1S, CDH1, CTGF, FN1, c-FOS, IGFBP5, JUN, LTBP1, LYN, S100A8, SOX4, SPP1, STC1, THBS1 and TNFAIP3. The interaction matrix showing the relation between CIGs and the included gene signatures is shown in **Figure 1**.

**Figure 2:**
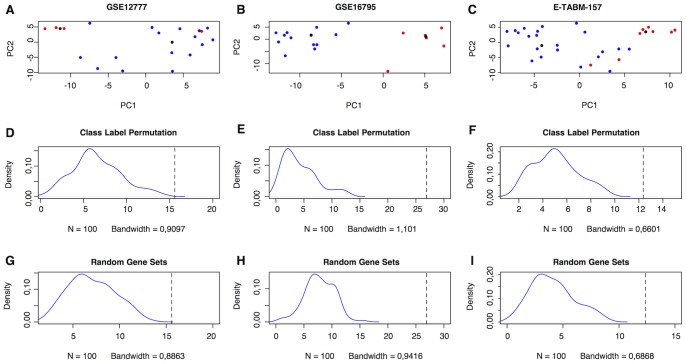
Validation of the CIG signature using breast cancer cell line gene expression data. The top row (A–C) shows the scatter plot representations of the PCAs performed on the distinct breast cancer cell lines data sets (GSE12777, GSE16795 and E-TABM-157) using the CIG signature. The X-axis represents the first principal component; the Y-axis represents the second principal component. A blue dot indicates a non-invasive breast cancer cell line and a red dot indicates an invasive breast cancer cells line. The black dots represent the centroids of the invasive and non-invasive cell lines. The middle row (D–F) shows the distributions of the Euclidean distances between the centroids of the invasive and non-invasive cell lines after class label permutation. The dashed vertical line indicates the true Euclidean distance between the centroids of the invasive and non-invasive cell lines. The lower row (G–I) represents the distributions of the Euclidean distances between the centroids of the invasive and non-invasive breast cancer cell lines based upon random selections of 16 genes from the group of 2636 genes obtained from the collection signatures associated with cell motility or invasion. The dashed vertical line indicates the Euclidean distance between the centroids of the invasive and non-invasive cells based on the 16 CIGs. These data demonstrate that the CIG signature is able to segregate invasive and non-invasive breast cancer cell lines and performs better then random selections of genes, which validates our gene selection strategy.

To validate the CIG signature we performed PCA on publicly available gene expression data sets of breast cancer cell lines (GSE12777, GSE16795, E-TABM-157). The Euclidean distance between the centroids of the invasive and the non-invasive breast cancer cells was respectively 15.587, 26.907 and 12.361. Class label permutation demonstrated that the observed Euclidean distances were significantly greater (all Ps<0.001) than the expected Euclidean distances (GSE12777: 6.575(6.016–7.134); GSE16795: 4.296(3.686–4.906) and E-TABM-157: 5.060(4.695–5.426)). Scatter plots and distributions of the expected Euclidean distances after class label permutation are displayed in **Figure 2**. To test whether the CIG signature performs better than sets of randomly selected genes in segregating the invasive and non-invasive cells, we performed PCA using 100 sets of 16 genes randomly selected from the list of 2636 unique genes represented by the original gene signatures included in this analysis. The average Euclidean distance between the centroids of the invasive and non-invasive cells for the randomly selected genes sets (GSE12777: 7.036(6.545–7.527); GSE16795: 7.814(7.293–8.336) and E-TABM-157: 4.078(3.697–4.456)) is significantly smaller than the Euclidean distance observed using the CIG signature (all Ps<0.001). Distributions of the random Euclidean distances are provided in **Figure 2**.

**Figure 3:**
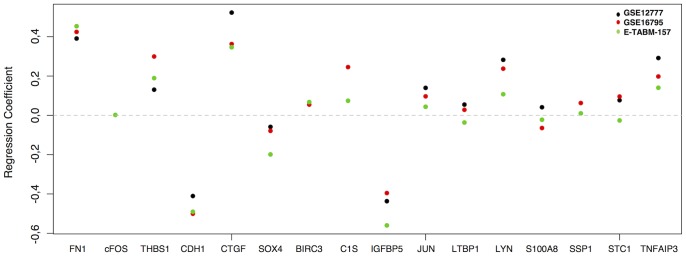
PCA for the CIGs on the breast cancer cell line data sets. Regression coefficients for the first principal components obtained by performing PCA for the CIGs on the breast cancer cell line data sets. The X-axis represents the 16 CIGs, the Y-axis represents the regression coefficients. The black, red and green dots are indicative for respectively GSE12777, GSE16795 and E-TABM-157. Positive and negative regression coefficients indicate respectively pro-invasive and contra-invasive genes. The magnitude of the regression coefficient reflects the importance of the corresponding gene in determining the CIG expression. The horizontal dashed line indicates a regression coefficient of zero. Some genes are represented less than 3 times due to the fact that not all CIG were amongst the informative gene list in every cell line data set. Most of the CIGs, except for S100A8, STC1 and LTBP1 show consistent regression coefficients indicating that they have a similar behaviour with respect to the prediction of the invasiveness phenotype of breast cancer cells in all 3 data sets.

To evaluate the robustness of the CIGs we compared the regression coefficients of the CIGs in the first principal components between the different data sets **(** **Figure 3** **)**. The regression coefficients in all 3 data sets show similar trends for both directionality (the sign of the regression coefficient) and amplitude (magnitude of the absolute value of the regression coefficient), except for STC1, S100A8 and LTBP1. The range of the regression coefficients for those genes crosses zero. For c-FOS, only 1 data point is reported as the gene was excluded from the list of informative genes in the remaining data sets (GSE12777 and GSE16795). Altogether, our data indicate that the greatest amount of variation in CIG expression resides in the difference between invasive and non-invasive breast cancer cells and that the set of CIGs are robust biomarkers to evaluate the invasive ability of tumour cells by gene expression analysis.

### Association between CIG Expression and EMT

**Figure 4:**
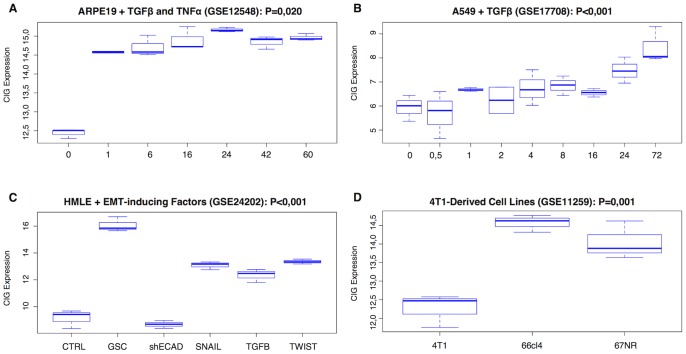
Boxplots showing the relation between CIG expression and EMT. The top row (A–B) represents a time series of different cell lines treated with EMT-inducing factors. These data demonstrate that CIG expression increases by incubation time. The lower left boxplot (C) indicates that CIG expression is induced by all of the known EMT-inducing factors, but most strongly downstream of GSC. The lower right boxplot (D) indicates that CIG expression does not necessarily correlate with metastatic capability as the cell line with the highest metastatic capability has the lowest CIG expression.

To evaluate the association between CIG expression and EMT we analysed 2 publicly available gene expression data sets of a time series of cancer cell lines treated with TGFβ alone (GSE17708) or in combination with TNFα (GSE12548). In both data sets we observed a significant differences in CIG expression with respect to the treatment time (Kruskal-Wallis: P<0.001 and P = 0.020 respectively). In addition, the CIG expression was positively correlated with the treatment time in both data sets (Spearman correlation: R_s_ = 0.757, P<0.001 and R_s_ = 0.514, P = 0.020 respectively). Results are displayed in **Figure 4A** and **Figure 4B**. These data indicate that the CIG expression increases in conditions with more pronounced EMT.

Next we analysed the expression of the CIG signature in function of the induction of EMT downstream of several EMT-inducing factors (TWIST, SNAIL, GSC and TGFβ) or loss of E-Cadherin expression (GSE24202). Again, significant between-group differences in CIG expression were observed (Kruskal-Wallis: P<0.001). Data are shown in **Figure 4C**. Post hoc testing demonstrated that CIG expression increased relative to the control level upon overexpression of TWIST (P<0.001), SNAIL (P<0.001), GSC (P<0.001) or TGFβ (P<0.001) to the HMLE cells. Silencing of E-Cadherin in HMLE cells did not significantly alter the level of CIG expression relative to the control level (P = 0.799). Interestingly, no significant differences (P>0.050) were observed for CIG expression upon overexpression of TWIST, SNAIL or TGFβ. However, CIG expression was significantly elevated upon overexpression of GSC relative to TWIST, SNAIL and TGFβ (All Ps<0.001).

Finally, we evaluated the significance of CIG expression in function of metastatic ability. Therefore we analysed the gene expression profiles of cell lines derived from a 4T1 tumour. The distinct 4T1-derived (4T1, 66cl4 and 67NR) cell lines exhibited different features with respect to EMT, *in vitro* invasiveness and *in vivo* metastatic ability. The CIG expression differed significantly between the three cell lines (Kruskal-Wallis: P = 0.001). Data are shown in **Figure 4D**. Post hoc testing revealed a significantly lowered expression in the 4T1 cells relative to the 66cl4 (P = 0.001) and the 67NR cells (P = 0.004). Interestingly, the 4T1 cells have the highest metastatic capacity although they do not express mesenchymal markers such as vimentin and N-Cadherin, whereas the 67NR cells have a mesenchymal phenotype but fail to metastasize. These data again demonstrate the association between EMT and CIG expression and also suggest that EMT as such may not be a prerequisite for elevated metastatic potential.

### Translational Significance in Breast Cancer

**Figure 5:**
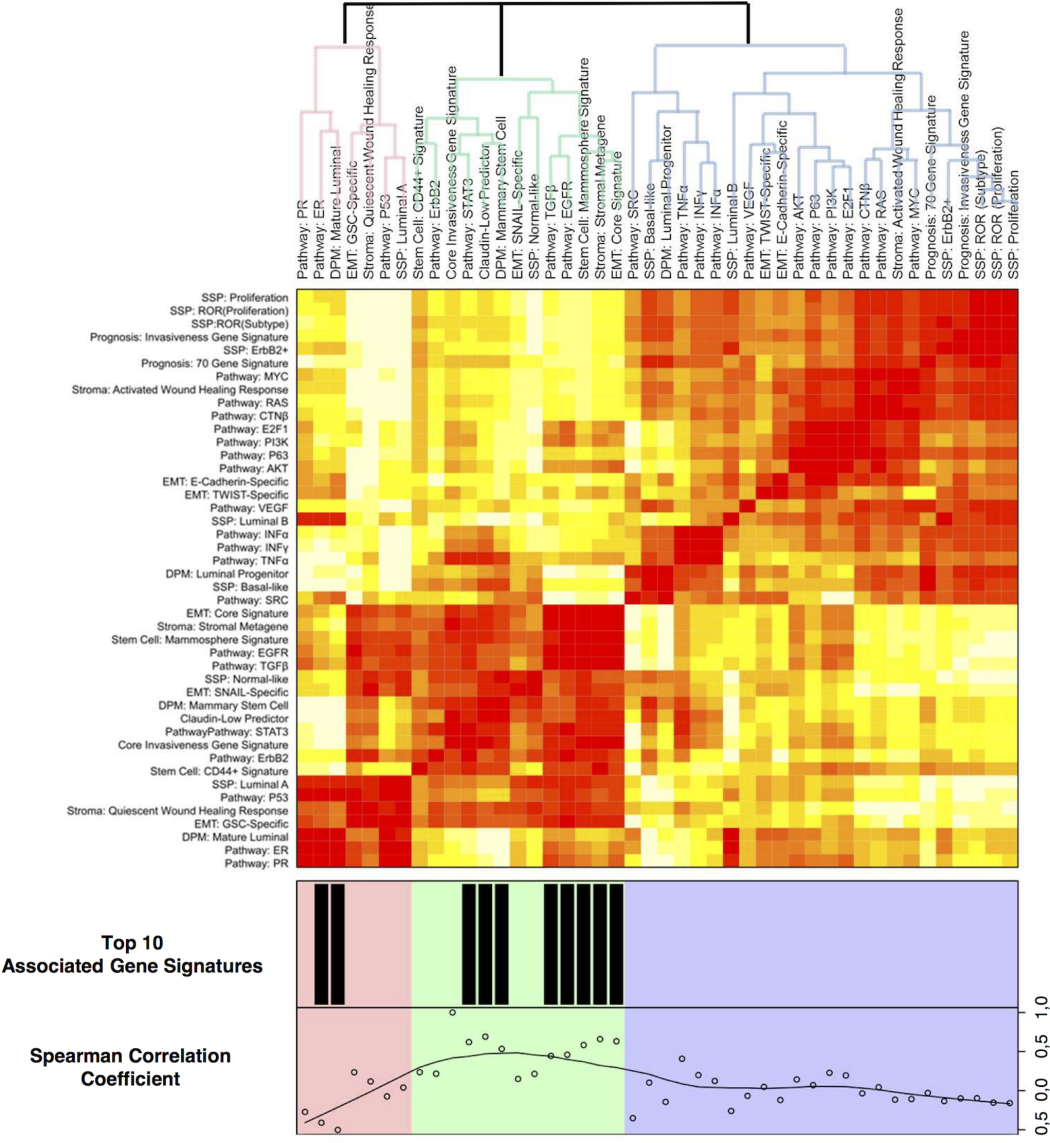
Association between published gene signatures and the CIG signature in human breast cancer. Heatmap showing the association between the expressions of several published gene signatures and the CIG signature in a set of approximately 2.500 breast tumour samples. The rows and columns represent the set of analysed gene expression signatures organized into groups related to prognosis, EMT, pathway activation, stem cell biology, breast tumour heterogeneity and stromal involvement. The cells at the intersection between the rows and the columns are colour-coded with red indicating a positive correlation between the respective gene signatures and white indicating a negative correlation. Colour saturation is associated the magnitude the correlation coefficient. The dendrogram is divided in 3 groups (red, blue and green) of strongly associated gene signatures. Underneath the heatmap the Spearman correlation coefficients between the CIG signature and the remaining signatures is represented as well as the ten signatures most strongly associated with the CIG signature.

Twelve gene expression data sets, vouching for a total of 2420 breast tumour samples, were retrieved from GEO. Each of the samples was classified according to a set of published gene signatures. In addition, we generated SNAIL, TWIST, GSC, and E-Cadherin specific gene signatures **(Figure S2–Figure S3)**. Unsupervised hierarchical clustering was performed on the classification scores (including CIG expression) and pathway activation scores. The resulting heatmap is shown in **Figure 5**. We observe 3 clusters linked to the differentiation status of breast cells: mature luminal cells, luminal progenitor cells and mammary stem cells. The cluster related to the mature luminal cells includes gene signatures associated with slowly proliferating ER+ breast tumours (Luminal A). Conversely, the cluster related to the luminal progenitor cells is predominated by gene signatures of highly proliferative tumours (Luminal B, Basal-like and ErbB2+) and includes the poor prognosis signatures (IGS, 70GENE, WHR, ROR_S and ROR_P). This observation agrees with the hypothesis that genes associated with cell proliferation are the main drivers of these signatures. As expected, the Luminal B gene signature is also associated with elevated ER signalling. The third cluster contains gene signatures that are associated with stem cell biology and incorporates most of the EMT-related gene signatures, including the one representing Claudin-low breast tumours. The EMT-related signatures do not reveal a coherent cluster pattern with TWIST- and E-Cadherin-specific signatures allocated to the luminal progenitor cell cluster, the TGFβ- and SNAIL-specific signatures allocated to the mammary stem cell cluster and the GSC-specific signature allocated to the mature luminal cell cluster. Of note is the hierarchy of the identified subgroups in the cluster dendrogram, which shows that the mammary stem cell cluster is more closely related to the mature luminal cell cluster and not the luminal progenitor cell cluster.

The CIG signature is contained in the EMT/mammary stem cell cluster, corroborating our view that CIG expression identifies breast tumour samples with a mesenchymal gene expression profile. To further elaborate on the biological significance of the CIG signature, we compared the classification scores of each signature with the CIG expression data. Due to large amount of samples, all P-values show at least a trend towards significance (P<0.1). The 10 most correlated gene signatures have correlation coefficients of at least 0.40 and clearly establish the relationship between CIG expression and EMT in breast cancer. Correlation coefficients are provided underneath the heatmap in **Figure 5**.

To associate CIG expression and EMT with metastatic potential in human breast cancer, we performed survival analysis relating all analysed gene signatures with Distant Metastasis Free Survival (DMFS). We included 1508 expression profiles of patients with breast cancer, from which 481 patients developed distant metastases. The median follow-up for patients with and without metastatic disease is 2.57 years and 8.11 years respectively. In univariate analysis, the CIG signature and 9 additional signatures were not associated with DMFS (P>0.050). Results are shown in **Table 3**. Using a 2-step multivariate analysis on the significant variables from univariate analysis, we identified 7 parameters that were independently associated with DMFS. The mammosphere signature (β = 0.597; P = 0.008) and the gene signatures associated with MYC- (β = 0.531; P<0.001), P53- (β = 0.682; P<0.001), TWIST- (β = 0.839; P<0.001), and SNAIL-activation (β = 0.863; P = 0.049) are associated with longer DMFS. The gene signatures for the Luminal B-phenotype (β = 1.771; P<0.001) and CD44+ breast tumour cells (β = 2.009; P<0.001) demonstrate the opposite pattern. When comparing the different datasets with respect to DMFS using Kaplan-Meier analysis, we found significant (P<0.001) dataset-specific differences. Results are shown in **Figure S4**. Therefore we decided to analyse each of the independent prognosticators in a multivariate model incorporating data set membership. All 7 variables retained their significance (P<0.05), indicating that the identified differences are not data set-specific.

**Table 3:** Survival analysis.

				UNIVARIATE				MODEL 1				MODEL 2			
	Parameter	N	Cluster	β value	CI-low	CI-high	P-value	β value	CI- low	CI-high	P-value	β value	CI-low	CI-high	P-value
**Molecular subtype**	Basal like	1508	Luminal Progenitor	1.996	1.621	2.459	P<0.001	–	–	–	–	–	–	–	–
	ErbB2+	1508	Luminal Progenitor	2.915	2.198	3.864	P<0.001	–	–	–	–	–	–	–	–
	Luminal A	1508	Luminal Mature Cell	0.398	0.326	0.486	P<0.001	0.525	0.391	0.707	P<0.001	–	–	–	–
	Luminal B	1508	Luminal Progenitor	1.431	1.117	1.832	P = 0.004	0.422	0.173	1.032	–	0.771	1.325	2.367	P<0.001
	Normal-like	1508	Mammary Stem Cell	0.486	0.379	0.623	P<0.001	0.310	0.117	0.822	–	–	–	–	–
	Claudin-Low	1508	Mammary Stem Cell	0.945	0.633	1.411	P = 0.782	–	–	–	–	–	–	–	–
**Prognostic signatures**	Proliferation (SSP)	1508	Luminal Progenitor	1.826	1.570	2.124	P<0.001	1.483	1.191	1.848	P<0.001	–	–	–	–
	70 Gene Prognostic Index	1508	Luminal Progenitor	2.758	2.124	3.581	P<0.001	1.661	1.132	2.438	–	–	–	–	–
	Invasiveness Gene Signature	1508	Luminal Progenitor	3.738	2.561	5.455	P<0.001	–	–	–	–	–	–	–	–
**Stem Cell signatures**	Mammosphere Signature	1508	Mammary Stem Cell	0.507	0.427	0.603	P<0.001	0.540	0.442	0.662	P<0.001	0.597	0.488	0.731	P<0.001
	CD44+ Signature	1508	Mammary Stem Cell	2.060	1.610	2.637	P<0.001	2.236	1.757	2.486	P<0.001	2.009	1.567	2.576	P<0.001
	Luminal Mature Cell (DPM)	1508	Luminal Mature Cell	0.523	0.362	0.756	P<0.001	–	–	–	–	–	–	–	–
	Luminal Progenitor Cell (DPM)	1508	Luminal Progenitor	3.903	2.598	5.863	P<0.001	1.724	1.258	2.808	–	–	–	–	–
	Mammary Stem Cell (DPM)	1508	Mammary Stem Cell	1.253	0.757	2.076	P = 0.381	–	–	–	–	–	–	–	–
**Stromal signatures**	Quiescent WHR	1508	Luminal Mature Cell	0.246	0.140	0.430	P<0.001	0.291	0.164	0.516	P<0.001	–	–	–	–
	Activated WHR	1508	Luminal Progenitor	3.559	2.133	5.939	P<0.001	–	–	–	–	–	–	–	–
	Stromal Metagene	1508	Mammary Stem Cell	0.766	0.681	0.862	P<0.001	0.800	0.710	0.903	P<0.001	–	–	–	–
**Pathway activation**	AKT	1508	Luminal Progenitor	0.727	0.611	0.865	P<0.001	0.638	0.492	0.827	P<0.001	–	–	–	–
**signatures**	CTNβ	1508	Luminal Progenitor	1.191	1.099	1.291	P<0.001	–	–	–	–	–	–	–	–
	E2F1	1508	Luminal Progenitor	0.916	0.846	0.992	P = 0.030	–	–	–	–	–	–	–	–
	EGFR	1508	Mammary Stem Cell	0.540	0.439	0.664	P<0.001	–	–	–	–	–	–	–	–
	ER	1508	Luminal Mature Cell	0.782	0.714	0.857	P<0.001	–	–	–	–	–	–	–	–
	ERBB2	1508	Mammary Stem Cell	0.998	0.904	1.102	P = 0.965	–	–	–	–	–	–	–	–
	INFα	1508	Luminal Progenitor	1.017	0.986	1.049	P = 0.278	–	–	–	–	–	–	–	–
	INFγ	1508	Luminal Progenitor	1.001	0.970	1.033	P = 0.949	–	–	–	–	–	–	–	–
	MYC	1508	Luminal Progenitor	1.236	1.030	1.483	P = 0.022	0.746	0.559	0.996	–	0.531	0.423	0.666	P<0.001
	P53	1508	Luminal Mature Cell	0.725	0.673	0.781	P<0.001	0.597	0.497	0.716	P<0.001	0.682	0.614	0.758	P<0.001
	PI3K	1508	Luminal Progenitor	0.882	0.809	0.963	P = 0.005	0.854	0.746	0.958	–	–	–	–	–
	PR	1508	Luminal Mature Cell	0.885	0.853	0.918	P<0.001	1.139	1.054	1.231	–	–	–	–	–
	RAS	1508	Luminal Progenitor	1.534	1.223	1.924	P<0.001	2.019	1.281	3.184	–	–	–	–	–
	SRC	1508	Luminal Progenitor	1.112	1.057	1.170	P<0.001	–	–	–	–	–	–	–	–
	STAT3	1508	Mammary Stem Cell	1.067	0.833	1.368	P = 0.607	–	–	–	–	–	–	–	–
	TNFα	1508	Luminal Progenitor	1.151	0.971	1.363	P = 0.105	–	–	–	–	–	–	–	–
	TGFβ	1508	Mammary Stem Cell	0.728	0.626	0.847	P<0.001	–	–	–	–	–	–	–	–
	VEGF	1508	Luminal Progenitor	1.189	1.032	1.369	P = 0.017	–	–	–	–	–	–	–	–
	P63	1508	Luminal Progenitor	0.943	0.859	1.035	P = 0.213	–	–	–	–	–	–	–	–
**EMT signatures**	EMT	1508	Mammary Stem Cell	0.940	0.922	0.959	P<0.001	0.971	0.948	0.995	–	–	–	–	–
	SNAIL	1508	Mammary Stem Cell	0.745	0.654	0.851	P<0.001	0.855	0.746	0.981	–	0.863	0.746	1.000	P = 0.049
	TWIST	1508	Luminal Progenitor	0.901	0.834	0.974	P = 0.008	0.811	0.742	0.885	P<0.001	0.893	0.774	0.907	P<0.001
	GSC	1508	Luminal Mature Cell	0.884	0.847	0.924	P<0.001	0.875	0.827	0.927	P<0.001	–	–	–	–
	E-CADHERIN	1508	Luminal Progenitor	1.025	0.989	1.063	P = 0.168	–	–	–	–	–	–	–	–
	Core Invasiveness Gene Signature	1508	Mammary Stem Cell	0.956	0.896	1.019	P = 0.168	–	–	–	–	–	–	–	–

## Discussion

In the present study we describe the identification of a set of biomarkers related to the invasive behaviour of (breast) cancer cells. We hypothesized that genes represented multiple times in a set of cell motility- and invasion-related gene lists have a higher propensity of being true biomarkers for the above-mentioned tumorigenic processes. We validated our signature by analysing 3 publicly available gene expression data on breast cancer cells, which were grouped according to their invasive potential using data published by Neve et al [25]. The robustness of the differential expression profile of the identified genes across all 3 data sets and the superior discriminative power to distinguish between invasive and non-invasive breast cancer cells with respect to random gene sets validates our hypothesis. Since we classified breast cancer cells according to their invasive potential, we named the identified gene list “Core Invasiveness Gene” signature.

Mining of gene functions associated with the core invasiveness genes suggests a tight link between CIG expression and EMT, a process in which tumour cells lose their epithelial phenotype to acquire a more mesenchymal phenotype. THBS1, FN1, CTGF and E-Cadherin are bona fide markers of EMT [52]. LYN is reported to be a top-ranked EMT signature gene and RNAi-mediated knockdown of LYN inhibited cell migration and invasion [53]. SPP1 is a member of a group of EMT-related genes identified by comparing the expression profiles of melanoma samples from patients with and without distant metastases [54]. The AP1-complex members c-JUN and c-FOS are involved in the activation of the promoter of MMP1 in MDA-MB-321 breast cancer cells secondary to the activation of ZEB1, a transcription factor involved in EMT [55]. In human immortal keratinocytes, EMT was induced by AP1-complexes downstream of TGFβ signalling [56]. The association between CIG expression and EMT was further explored through a set of *in silico* experiments. We observed that CIG expression increases in cell lines as they were treated for increasing amounts of time with EMT-inducing factors. Also, overexpression of SNAIL, TWIST, GSC and TGFβ in HMLE cells led to augmented CIG expression relative to the control or mock-transfected conditions. Surprisingly, knockdown of E-Cadherin in the same cell line did not result in augmented CIG expression, although E-Cadherin is included in the list of core invasiveness genes. Finally, the analysis of transcriptional profiles of about 2500 breast tumour samples revealed that CIG expression in human breast cancer is associated EMT-related features and with the Claudin-low phenotype, a breast cancer subtype characterized by the elevated expression of mesenchymal markers [28] [42].

One of the goals to embark on the quest of identifying biomarkers associated with increased invasiveness was the premise that such biomarkers could aid in identifying patients at risk of development of distant metastases. In contrast to our expectations, we found that CIG expression or the presence of EMT-associated features does not correlate with metastatic potential. CIG expression was lowered in highly metastatic clones derived from the murine 4T1 breast cancer cell line. The analysis of CIG expression in function of the development of distant metastases demonstrated that patients with elevated CIG expression levels do not exhibit a poor prognosis profile. The analysis of other EMT-associated gene signatures in this study supported this finding and corroborates previous studies that failed to demonstrate a link between EMT and metastatic potential [28] [44]. On the other hand, several studies did report that EMT was associated with the absence of a (complete) pathological response to neoadjuvant chemotherapy [28] [44], which can be explained by the fact that tumour cells undergoing EMT acquire a stem cell phenotype [57]–[58]. Also in this study, we observed that tumour samples with elevated expression levels of EMT-related features exhibit stem cell characteristics.

In general, our data seem to suggest that EMT-like invasive tumour cell behaviour is not required for successful metastasis. Nevertheless, several points need to be considered prior to taking this conclusion for granted. First, the invasive behaviour might reside in a small fraction of tumour cells. Therefore, the contribution of these cells to the global gene expression profile of breast tumour samples is limited which might obscure their association with the development of distant metastases. This hypothesis however does not apply to the expression data obtained from the clones of the murine 4T1 cell line and therefore will not provide the sole explanation for our observations. Second, the acquisition of an mesenchymal phenotype might not be sufficient to capture the metastatic potential. Fibroblasts for example, which are characterized by a mesenchymal expression profile [59], do not metastasize. In addition to becoming invasive, tumour cells need to be able to disseminate and survive in the blood stream in order to spread to distant organs. The role of angiogenesis, and by extension the tumour host, in the metastatic cascade should also be considered.

In spite of the above, the most likely hypothesis states that EMT as such is not a prerequisite for successful metastasis. Several lines of evidence obtained in this study support this view. The 4T1-clone 67NR has low expression of E-Cadherin and elevated expression of vimentin and N-Cadherin. CIG levels in this cell line were significantly elevated when compared to the parental 4T1 cell line, which expresses E-Cadherin and is characterized by a more epithelial phenotype. Nevertheless, the metastatic potential of the 4T1 cell line is far superior, indicating that EMT is not always necessary for the formation of metastases. A parallel can be drawn between the observations made on the 4T1 clones and the situation observed in inflammatory breast cancer (IBC). IBC is an aggressive subtype of locally advanced breast cancer with a significant degree of local invasion and distant metastasis [60]. Tumour cells from patients with IBC often express E-Cadherin [61]–[63], which has been regarded as a paradox due to the high metastatic nature of IBC. In this context, we observed that EMT is not more pronounced in IBC as compared to non-IBC breast cancer samples *(data not shown)*. Studies on the SUM149 IBC cell line actually demonstrated that the invasive nature of IBC tumour cells critically depends on the overexpression of functional E-Cadherin and the influence thereof on MMP1 and MMP9 expression [64]. Another intriguing observation, made by Giampieri and colleagues [65], suggests that reduced levels of TGFβ, a negative regulator of E-Cadherin through SNAIL and TWIST [66]–[67], prevent tumour cells from moving individually but do not inhibit cells moving collectively. Moreover, cells moving collectively were capable of lymphatic invasion but not blood-borne metastasis. Thus, lowered TGFβ levels would allow tumour cell clumps to home to the lymphatic system. This view can be easily translated into the pathological hallmark of IBC, namely tumour emboli in the dermal and parenchymal lymph vessels [68]. In our data, reduced SNAIL activation is an independent predictor of the IBC phenotype and gene expression data did suggest that TGFβ-activation in IBC is indeed lowered (*data not shown).*

A last point that needs to be considered with respect to this study relates to the fact that EMT is an extremely dynamic process, governed by a plethora of transcription factors. The acquisition of a mesenchymal morphology is the end-point of EMT but the routes towards the end-point might differ between conditions and cells. Therefore, different EMT-signatures might represent different flavours of EMT, driven by alternative pathways, and conclusions with respect to EMT based on only one EMT-related signature should be made with care. In light of this statement, we observed a significant overexpression in IBC of 2 CIGs, associated with the NFκB pathway. The NFκB transcription factor has been associated with EMT [69] and previous studies have shown that NFκB is an important molecular characteristic of IBC [70]–[71].

In conclusion, the data presented in this paper add to the discussion related to the importance of invasion and EMT for the development of distant metastases. Given the concerns discussed above, a definitive conclusion cannot be drawn. However, our data do show that a clear and positive relation between EMT and metastatic potential is not readily observable. In fact, our data suggest that the opposite might be true, although the magnitude of the hazard ratios requests caution. Specifically due to the large amounts of samples analysed in this paper, small but biologically irrelevant differences can become significant. Whether these observations apply only to EMT or can be extended to other types of invasion (e.g. collective invasion) remains unclear. Either way, our data do put forward a list of research question that warrant further investigation.

## Supporting Information

Figure S1 **Identification of genes differentially expressed in response to Ezrin knockdown or RhoA activation.** To identify genes associated with knockdown of Ezrin, a critical regulator of the actin cytoskeleton we downloaded data set GSE11279. Raw expression data were normalized using the frozen RMA algorithm and probe sets with fluorescence intensities above log2(100) in at least 10% of the cases were filtered in. Using significance analysis of microarrays (SAM) we identified differentially expressed genes between SW480 cells treated with and without siRNA against Ezrin. Due to the small sample size (N = 4) we decided to use a δ-value corresponding to a false discovery rate (FDR) of 10% resulting in 31 significant probe sets. The corresponding SAM-plot is provided in (A). The list of 31 probe sets corresponded to 26 unique genes. This list was included in the collection of cell motility and invasion related gene lists used for the overrepresentation analysis. To identify genes associated with activation of RhoA, a critical regulator of the cell motility via its function in modulating the actin cytoskeleton we downloaded data set GSE12917. Data were preprocessed as described before. Using SAM we identified differentially expressed between normal HMECs and HMECs transfected with RhoAG14V, a constitutively active mutant of RhoA. A δ-value was chosen as such that the FDR was less than 5%, resulting in 170 significant probe sets. The corresponding SAM-plot is provided in (B). The list of 170 probe sets corresponded to 135 unique genes. This list was included in the collection of cell motility and invasion related gene lists used for the overrepresentation analysis.
(TIF)

Figure S2 **Generation of the TWIST, SNAIL, GSC and E-Cadherin activation signatures.** We retrieved data set GSE24202 from the GEO-repository. Data preprocessing was done as described earlier. Using SAM we identified differentially expressed probe sets associated with each transcription factor by performing pair-wise comparison between the transfected and non-transfected conditions. A δ-value was chosen as such that the FDR was less than 5%. The resulting SAM-plots for each comparison are shown in (A–D). The corresponding δ-values and the number of genes called significant are reported with each SAM-plot. Next, we intersected the gene lists to identify genes that are specific only to one condition. As such we identified 141, 162, 993 and 845 genes that are respectively SNAIL-, TWIST-, GSC- and E-Cadherin-specific. Using these gene lists we performed principal component analysis to investigate whether the shrunken gene lists were still able to distinguish between the transfected and the non-transfected conditions. 2D scatter plot representations of the PCAs are shown in (E–H). For each EMT-inducing factor we observed a significant segregation of the transfected and the non-transfected conditions along the X-axis, which represents the first principal component. The regression coefficients responsible for the construction of the first metagene expression retrieved from each PCA were used to calculate the activation scores on novel data sets.
(TIF)

Figure S3 **2D scatter plot representation of the PCA on GSE24202 data set of the core-EMT signature.** In addition to the gene lists for the individual EMT-inducing factors, we retrieved the gene list for the core-EMT signature described by Taube et al (PNAS, 2010). This signature consists of all genes commonly deregulated by SNAIL, TWIST, GSC, E-Cadherin and TGFβ. We applied this gene signature onto its original data set (GSE24202) using PCA. The regression coefficients responsible for the construction of the first metagene expression retrieved from the PCA were used to calculate the EMT score on novel data sets.
(TIF)

Figure S4 **Survival analysis.** Due to the fact that the different data sets used throughout this study involve series of patient samples with differences in their clinicopathological characteristics, we first analysed data-set specific differences in DMFS. Using Kaplan-Meier analysis we identified significant data set-specific differences (P<0.001). The resulting Kaplan-Meier plot is demonstrated in supplementary figure 3. The most dramatic difference was observed for the data set GSE25055 (β = 19.961, 95%C.I. = 13.455–29.615). Due to this difference in survival, we incorporated the data set membership in the survival analysis to test whether the identified significant associations are data set-dependent.
(TIF)
