# Supplementary figures and images for: A Core Invasiveness Gene Signature Reflects Epithelial-to-Mesenchymal Transition but Not Metastatic Potential in Breast Cancer Cell Lines and Tissue Samples
Source: PLoS One. 2014 Feb 21;9(2):e89262. doi: 10.1371/journal.pone.0089262 (PMC3931724; doi:10.1371/journal.pone.0089262)

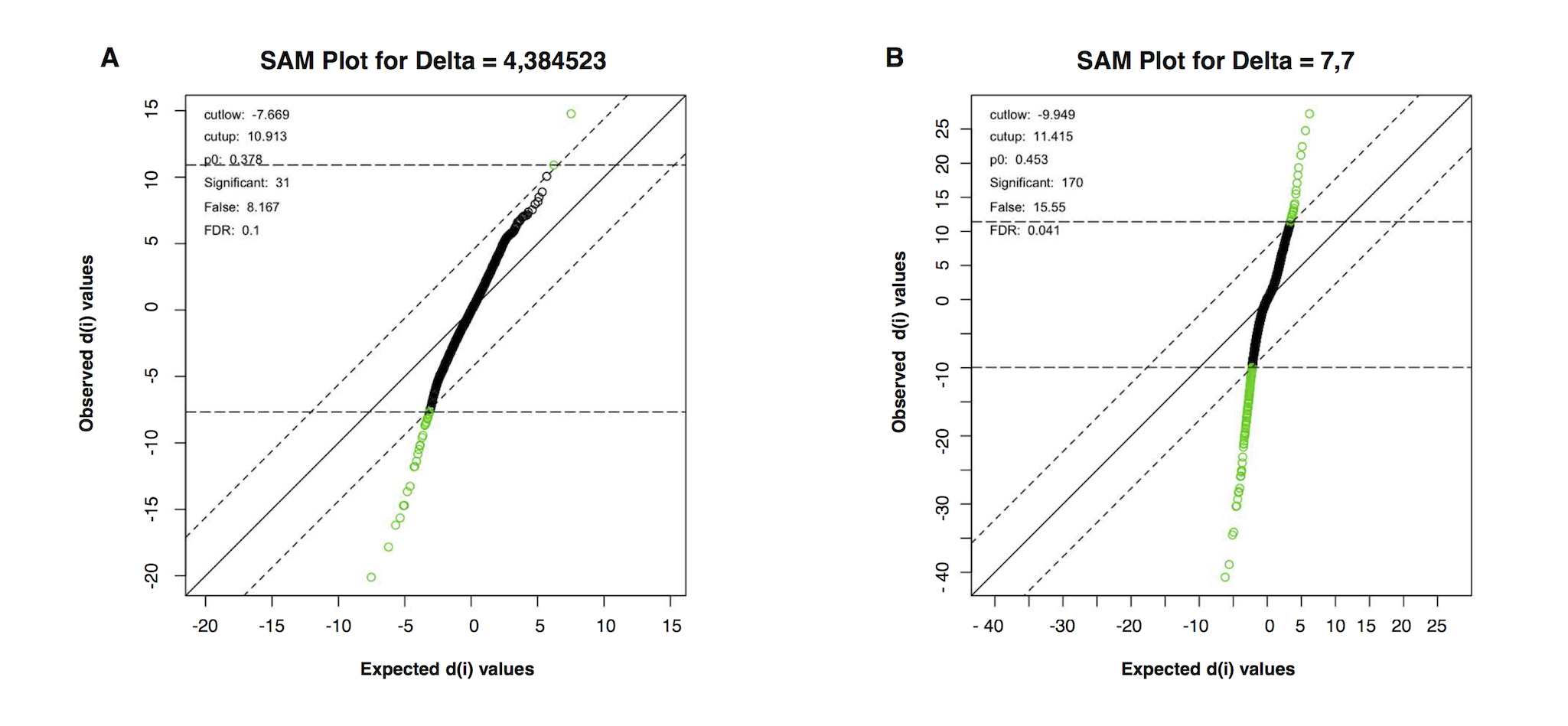

Supplement: Figure S1 — Identification of genes differentially expressed in response to Ezrin knockdown or RhoA activation. To identify genes associated with knockdown of Ezrin, a critical regulator of the actin cytoskeleton we downloaded data set GSE11279. Raw expression data were normalized using the frozen RMA algorithm and probe sets with fluorescence intensities above log2(100) in at least 10% of the cases were filtered in. Using significance analysis of microarrays (SAM) we identified differentially expressed genes between SW480 cells treated with and without siRNA against Ezrin. Due to the small sample size (N = 4) we decided to use a δ-value corresponding to a false discovery rate (FDR) of 10% resulting in 31 significant probe sets. The corresponding SAM-plot is provided in (A). The list of 31 probe sets corresponded to 26 unique genes. This list was included in the collection of cell motility and invasion related gene lists used for the overrepresentation analysis. To identify genes associated with activation of RhoA, a critical regulator of the cell motility via its function in modulating the actin cytoskeleton we downloaded data set GSE12917. Data were preprocessed as described before. Using SAM we identified differentially expressed between normal HMECs and HMECs transfected with RhoAG14V, a constitutively active mutant of RhoA. A δ-value was chosen as such that the FDR was less than 5%, resulting in 170 significant probe sets. The corresponding SAM-plot is provided in (B). The list of 170 probe sets corresponded to 135 unique genes. This list was included in the collection of cell motility and invasion related gene lists used for the overrepresentation analysis. (TIF) [file pone.0089262.s001.tif]

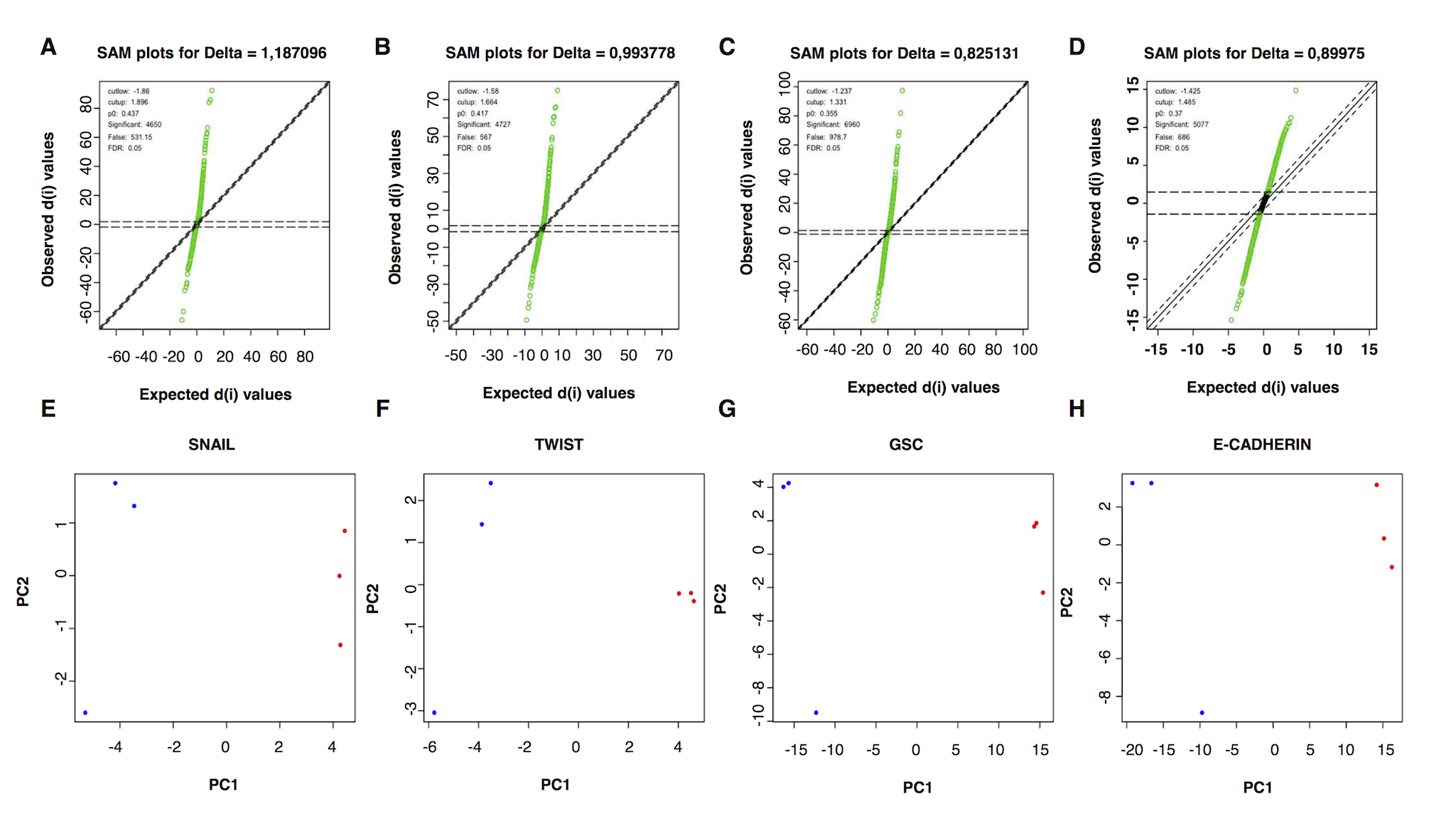

Supplement: Figure S2 — Generation of the TWIST, SNAIL, GSC and E-Cadherin activation signatures. We retrieved data set GSE24202 from the GEO-repository. Data preprocessing was done as described earlier. Using SAM we identified differentially expressed probe sets associated with each transcription factor by performing pair-wise comparison between the transfected and non-transfected conditions. A δ-value was chosen as such that the FDR was less than 5%. The resulting SAM-plots for each comparison are shown in (A–D). The corresponding δ-values and the number of genes called significant are reported with each SAM-plot. Next, we intersected the gene lists to identify genes that are specific only to one condition. As such we identified 141, 162, 993 and 845 genes that are respectively SNAIL-, TWIST-, GSC- and E-Cadherin-specific. Using these gene lists we performed principal component analysis to investigate whether the shrunken gene lists were still able to distinguish between the transfected and the non-transfected conditions. 2D scatter plot representations of the PCAs are shown in (E–H). For each EMT-inducing factor we observed a significant segregation of the transfected and the non-transfected conditions along the X-axis, which represents the first principal component. The regression coefficients responsible for the construction of the first metagene expression retrieved from each PCA were used to calculate the activation scores on novel data sets. (TIF) [file pone.0089262.s002.tif]

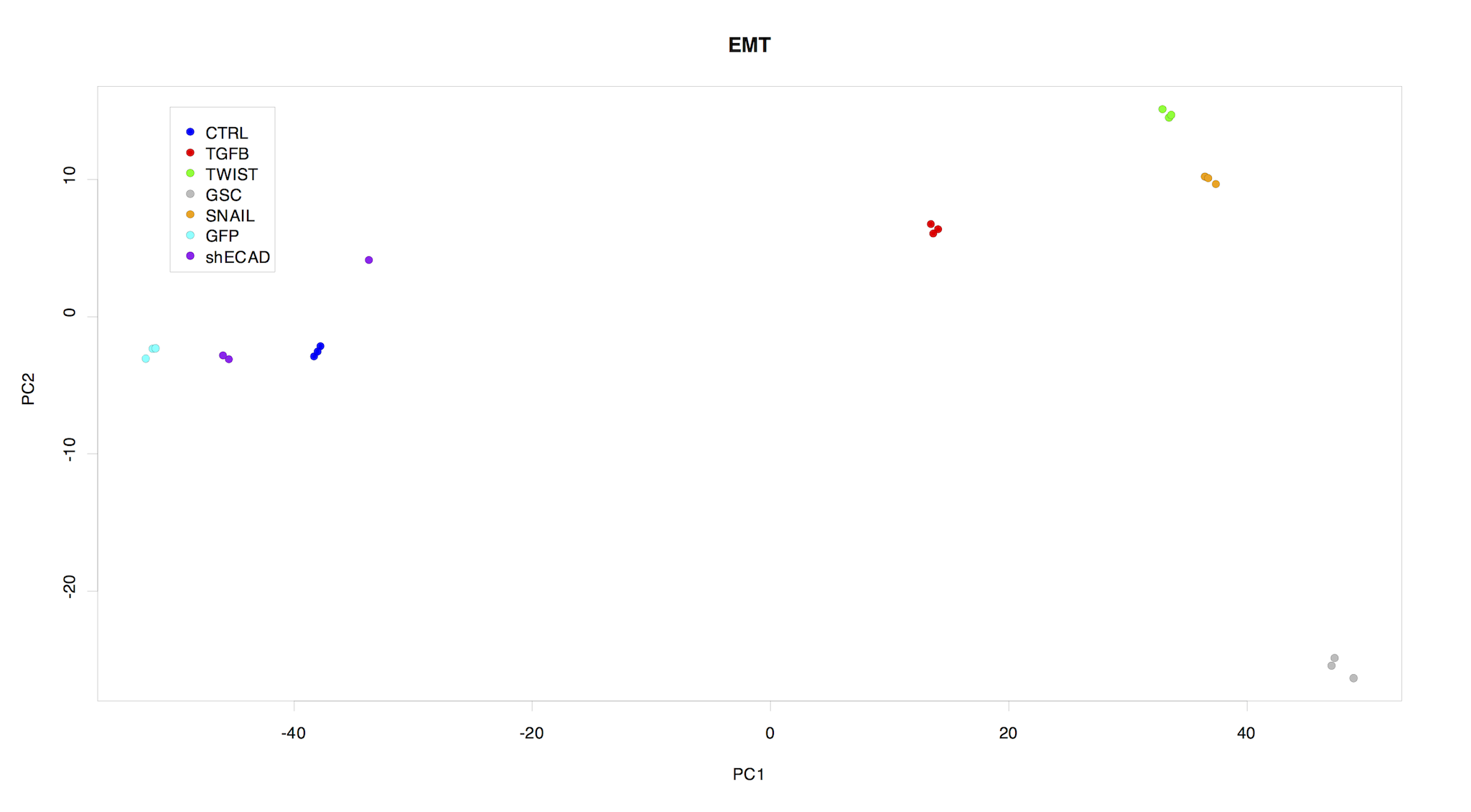

Supplement: Figure S3 — 2D scatter plot representation of the PCA on GSE24202 data set of the core-EMT signature. In addition to the gene lists for the individual EMT-inducing factors, we retrieved the gene list for the core-EMT signature described by Taube et al (PNAS, 2010). This signature consists of all genes commonly deregulated by SNAIL, TWIST, GSC, E-Cadherin and TGFβ. We applied this gene signature onto its original data set (GSE24202) using PCA. The regression coefficients responsible for the construction of the first metagene expression retrieved from the PCA were used to calculate the EMT score on novel data sets. (TIF) [file pone.0089262.s003.tif]

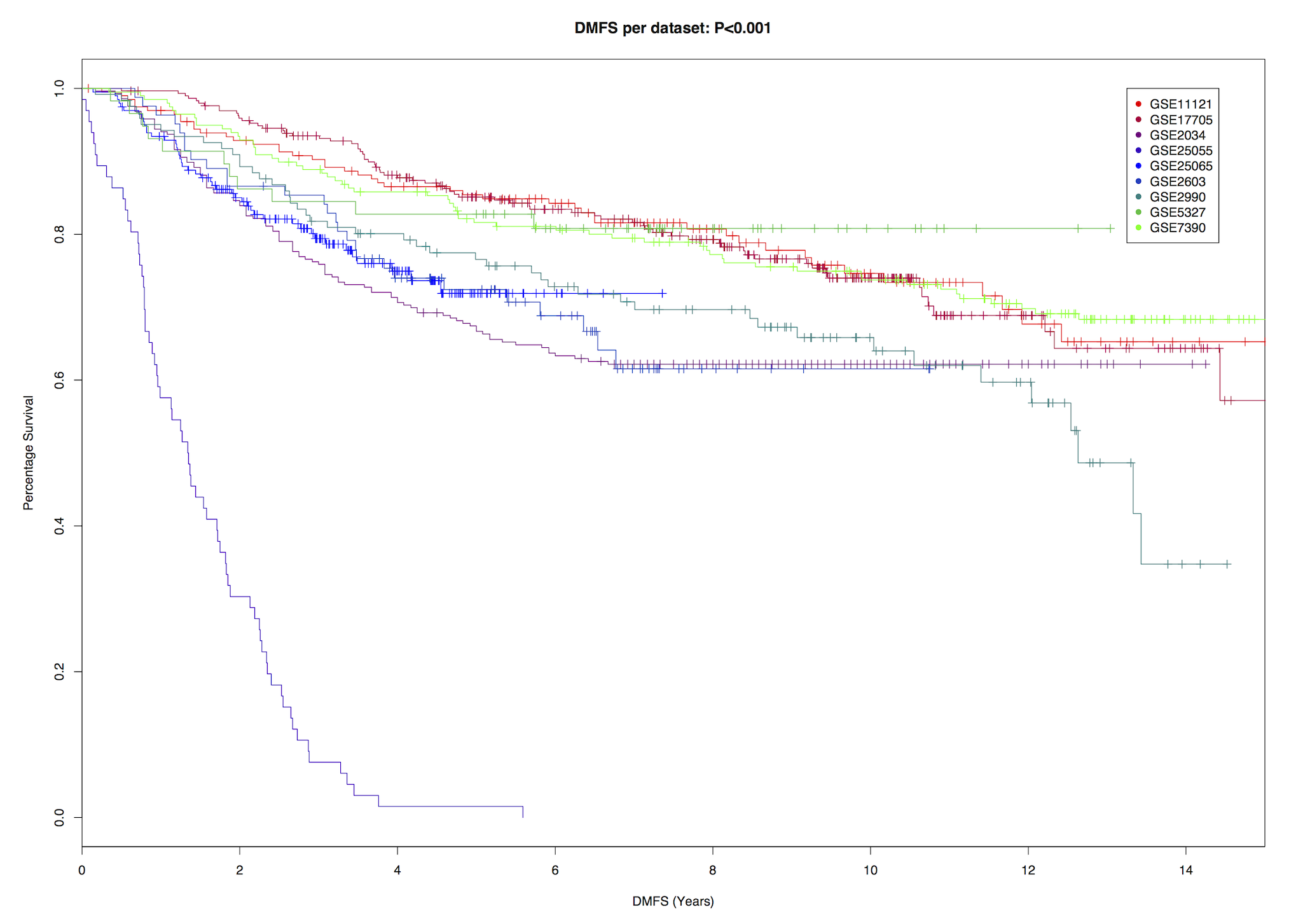

Supplement: Figure S4 — Survival analysis. Due to the fact that the different data sets used throughout this study involve series of patient samples with differences in their clinicopathological characteristics, we first analysed data-set specific differences in DMFS. Using Kaplan-Meier analysis we identified significant data set-specific differences (P<0.001). The resulting Kaplan-Meier plot is demonstrated in supplementary figure 3. The most dramatic difference was observed for the data set GSE25055 (β = 19.961, 95%C.I. = 13.455–29.615). Due to this difference in survival, we incorporated the data set membership in the survival analysis to test whether the identified significant associations are data set-dependent. (TIF) [file pone.0089262.s004.tif]
